# Draft Metagenome-Assembled Genomes from Methane-Rich Echo Lake, Montana

**DOI:** 10.1128/mra.01112-21

**Published:** 2022-02-03

**Authors:** Abigail M. Ross, Logan M. Peoples, Evan M. Bilbrey, Matthew J. Church

**Affiliations:** a Flathead Lake Biological Station, University of Montana, Polson, Montana, USA; University of Delaware

## Abstract

Five metagenome-assembled genomes were obtained from the bottom waters of Echo Lake, Montana. These genomes suggest that lineages involved in methane oxidation and sulfur cycling flourish near the steep oxygen and methane chemocline in Echo Lake.

## ANNOUNCEMENT

**FIG 1:**
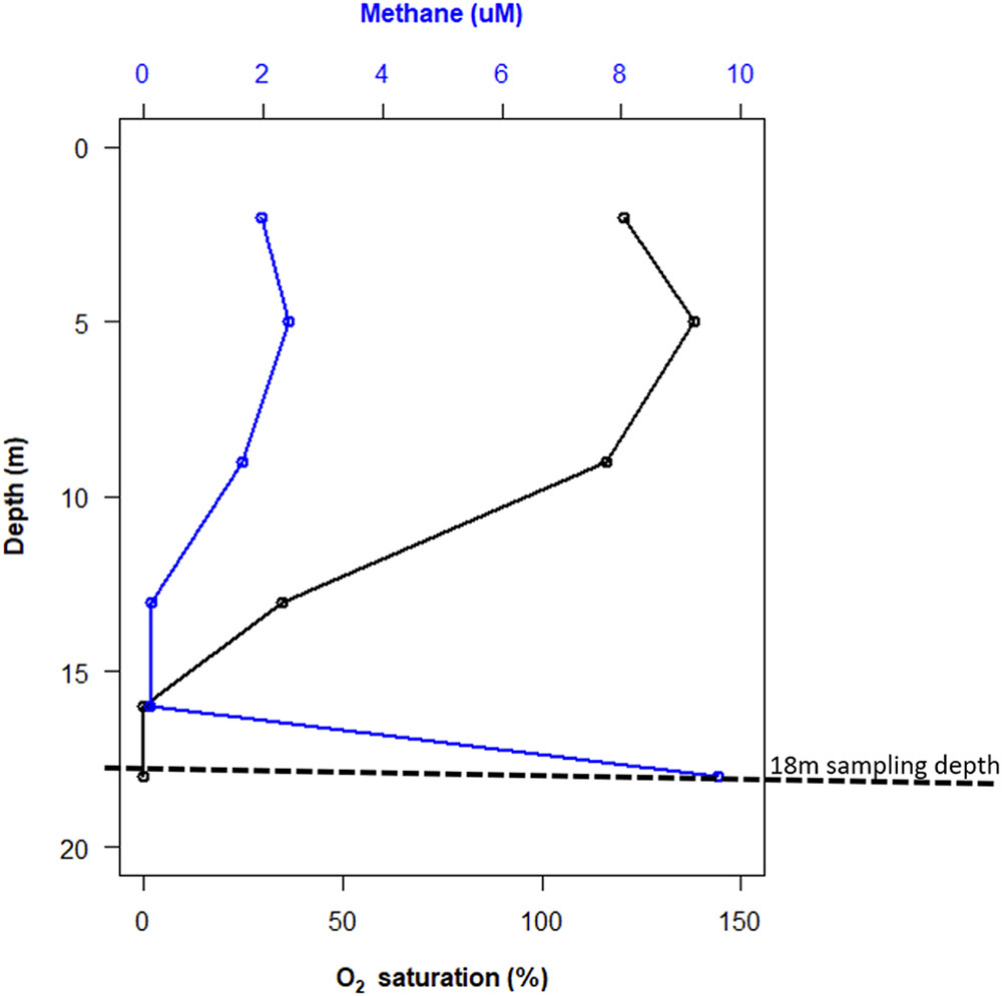
Depth profile of methane concentrations in Echo Lake, Montana, collected 22 July 2021. High methane concentrations were measured at the deepest depths within the anoxic zone. Discrete water samples for methane analyses were collected using a Van Dorn bottle, subsampled into crimp-sealed serum bottles, and amended with 8 M NaOH. Methane concentrations were determined by gas chromatography (model 8610C; SRI Instruments). Oxygen concentrations were measured using a Hydrolab (OTT HydroMet).

Echo Lake is a groundwater-fed pothole lake in the Flathead Valley in northwest Montana (1, 2). The lake has no outlet, leading to nutrient accumulation and anoxic bottom waters. A methane chemocline is present near the oxic/anoxic boundary (Fig. 1), indicating rapid consumption of methane in the water immediately overlying the bottom waters and sediments. We investigated the microbial community associated with this strong methane gradient by performing metagenomic sequencing.

Water was collected from Echo Lake (48.1228N, 114.0360W) on 10 July 2018. Samples were obtained from a depth of 18 m, 3 m above the bottom, using a discrete-depth Van Dorn bottle. The temperature upon collection was 4.5°C. Samples were stored in a cooler prior to processing in the laboratory. Approximately 1 L was filtered onto a 25-mm-diameter, 0.2-μm polyethersulfone filter (SUPOR; Pall Co., NY, USA), which was stored at −80°C. Genomic DNA was extracted using a MasterPure DNA purification kit (Lucigen, WI, USA). DNA libraries were prepared using a DNA preparation kit (Illumina, San Diego, CA, USA), and 150-bp paired-end reads were sequenced on a NextSeq 2000 system at the Microbial Genome Sequencing Center (MiGS) (Pittsburgh, PA). The number of raw reads obtained was 13,360,752. Raw reads were quality trimmed using Trimmomatic v0.39 (3) with the parameters LEADING:3 TRAILING:3 SLIDINGWINDOW:4:15 MINLEN:125. Trimmed reads were assembled using metaSPAdes v3.14.1 (4) using default parameters. The depth of coverage of the assembled contigs was estimated using Bowtie2 v2.3.5.1 (5) and SAMtools v1.10 (6). Genome bins were obtained using MetaBAT 2 v2.11.1 (7), with contigs of >5 kb being retained. The size and quality of each genome bin were evaluated using QUAST v5.0.2 (8) and CheckM v1.0.13 (9) with the --reduced_tree flag. We report genome bins with >50% completeness and <10% contamination, representing medium-quality draft genomes (10). Genomes were named taxonomically using GTDB-tk (11), and closely related strains were identified using orthoANIu (12). General features of each genome can be found in Table 1. Functional annotation was performed using Prokka v1.14.6 (13), GhostKOALA (14), KeggDecoder (15), and the NCBI Prokaryotic Genome Annotation Pipeline (PGAP) (16).

**TABLE 1:** Genomic features of the five metagenome-assembled genomes obtained from Echo Lake

Parameter	Data for genome for:				
	*Rhodoferax* sp. strain Echo1	*Chlorobium* sp. strain Echo2	*Methylotenera* sp. strain Echo3	*Methylovulum* sp. strain Echo5	“*Candidatus* Contendobacter” sp. strain Echo7
Completeness (%)	82.44	95.88	69.31	74.88	78.21
Contamination (%)	0.76	0	0	0.01	0.65
Coverage (×)	26	17	11	9	9
Length (Mbp)	3.07	2.17	1.25	1.99	2.69
No. of contigs	248	128	83	173	233
*N*_50_ (bp)	14,913	19,992	18,723	13,127	13,745
GC content (%)	60.38	47.85	49.5	41.33	58.36
No. of genes	2,946	2,101	1,270	1,888	2,507
NCBI assembly accession no.	GCA_020035295.1	GCA_020035305.1	GCA_020035195.1	GCA_020035215.1	GCA_020035205.1
Related strain (NCBI assembly accession no., ANI)	*Comamonadaceae* bacterium PowLak16_MAG17 (GCA_007280205.1, 98.44)	Pelodictyon phaeoclathratiforme BU-1 (GCA_000020645.1, 82.08)	*Methylotenera* sp. strain Baikal-deep-G82 (GCA_009693125.1, 74.47)	*Methylococcaceae* bacterium PowLak16_MAG1 (GCA_007280895.1, 99.15)	*“Candidatus* Competibacteraceae” bacterium CPB_P15 (GCA_003989085.1, 81.16)

The presence of microbes related to *Chlorobium*, *Methylotenera*, and *Rhodoferax* within Echo Lake is consistent with communities in other old, stratified lakes in which sulfur cycling and methylotrophy is prevalent (17). The genome related to the genus *Methylovulum* contains both particulate and soluble methane monooxygenase genes; this suggests that this genus is a major contributor to the steep methane chemocline and plays a vital role in regulating methane efflux from Echo Lake. No methanogens were found in the metagenome, suggesting that methane was likely produced in the sediments or introduced with groundwaters. Hence, future work should differentiate these potential sources of methane to the near-bottom waters of the lake.

### Data availability.

This genome sequencing project has been deposited in GenBank under the BioProject accession number PRJNA761446. The raw reads are available under the SRA accession number SRR15811987.
